# Development of lifestyle assessment: A Delphi survey of multi-faceted health experts

**DOI:** 10.1371/journal.pone.0316597

**Published:** 2025-02-19

**Authors:** Ah-Ram Kim, Young-Myoung Lim, Ji-Hyuk Park

**Affiliations:** 1 Yonsei New-Normal Lifestyle Research Center, Yonsei University, Wonju-si, South Korea; 2 Department of Occupational Therapy, College of Software and Digital Healthcare Convergence, Yonsei University, Wonju-si, South Korea; Public Library of Science, UNITED STATES OF AMERICA

## Abstract

**Objective:**

In the field of health promotion, there’s a growing focus on adopting an academic approach to assess and evaluate the intricate and ever-evolving nature of lifestyles. This approach is crucial for shaping and improving a healthy way of life. Systematic measurement of lifestyles is essential for enhancing overall health and well-being, encompassing physical, mental, and social dimensions. This study’s goal is to create an assessment tool that measures the diversity and intensity of lifestyle behaviors linked to human health. Our efforts involved developing quantitative measurement items that encompass the holistic concepts of health and lifestyle and validating them.

**Methods:**

Between March and April 2023, we gathered insights from 14 experts in lifestyle and health. Building on previous research, we conducted a Delphi survey twice. In the initial survey, we collected expert opinions through open and closed-ended questions about lifestyle evaluation items. After the first survey, we corrected several items that didn’t meet the Content Validity Ratio (CVR) standard before proceeding. We presented the first survey’s findings to an expert panel, leading to a consensus.

**Results:**

The initial Delphi round produced 76 items, and the second round resulted in 72 items after adjusting those with CVR scores of 0.51 or less. The final Content Validity Ratio was 0.83, signifying a robust validation process, with a convergence of 0.50 and a consensus level of 0.75.

**Conclusion:**

In summary, this study successfully developed a comprehensive lifestyle assessment tool using a modified Delphi technique. The 72 items are categorized into eight subcategories and four overarching themes. This tool provides a systematic approach to evaluate lifestyles, promoting health and well-being across physical, mental, and social dimensions. It also assists in identifying specific lifestyle elements that require more in-depth intervention strategies, all while preserving the academic integrity of the content.

## Introduction

Lifestyle is becoming a major subject in the development of academic meaning and socioeconomic aspects in fields such as health promotion, marketing, and sociology, focusing on human behavior [1–3]. Lifestyle is defined as a lifestyle formed according to external environmental factors that reflect an individual’s life consciousness, values, and personality while consuming time and money [4]. Lifestyles that reflect such multi-faceted aspects are being studied to identify the characteristics and trends of society, groups, and individuals or small groups [5].

Recently in the field of health promotion, interest in lifestyle has increased as it increases life expectancy while reducing the prevalence of chronic diseases, premature mortality, and non-communicable diseases [6,7]. Such a lifestyle is reported to be an important determinant of health and well-being in human life, and the need for a healthy lifestyle approach has been emphasized (WHO, 2021). Recently, an academic approach to lifestyle, which determines health and well-being, has been used in the analysis of causal relationships, risk factors, interventions, and the development of evaluation tools [3,8,9].

In the field of health promotion, the importance of an academic approach that can measure and evaluate a multi-dimensional and dynamic lifestyle has been emphasized to form and improve a healthy lifestyle [10]. Previous studies have quantified the frequency and exposure of behaviors such as physical activity, smoking, and drinking to analyze lifestyle characteristics; however, tools that can measure habits, behaviors, management, and diversity in daily life are limited [11]. Such a limitation makes it impossible to grasp the practice trend of a lifestyle that reflects the intensity of health-related routines and habits repeated in daily life and the diversity of activities [12].

Previous studies have focused on behaviors such as obesity, physical activity, diet, drinking, and smoking as lifestyle factors for human health and well-being [13,14]. These factors have previously been emphasized in the healthcare field [15]. On the other hand, the importance of relationships and participation in social factors has been emphasized before [16], but the need for a comprehensive approach that includes them as key relatively unnoticed factors is increasing [16,17].

Considering this research situation, it is essential to systematically measure lifestyles to achieve health and physical, mental, and social well-being [18]. However, no evaluation tool can measure the diversity and degree of a comprehensive healthy lifestyle in daily life, and the need for development has been confirmed. This study aims to develop an assessment tool to measure the diversity and degree of lifestyle behaviors related to human health. Therefore, we attempted to develop quantitative measurement items that reflect the comprehensive concepts of health and lifestyle and to verify their validity.

## Methods

The Delphi technique, developed by Dalkey and Helmer (1963) [19] at the Rand Corporation in the 1950s [20], is designed to gather purposeful opinions from experts identified in each field [21]. We used the modified Delphi method, which obtains consensus on the opinions of a group of experts through a series of structured open and closed questions [22]. The study was approved by the Yonsei University Mirae Institutional Review Board, considering the ethical aspects of the participants (Approval number:1041849-202211-SB-207-02). The data collection for this study was conducted from March 1, 2023, to April 30, 2023. Also, this study was conducted through an online survey, and given the impracticability of obtaining written consent, it proceeded after obtaining approval from the research ethics committee for an exemption from written consent.

### The expert panel

We aimed to recruit healthcare professionals who are considered experts in lifestyle and health. To this end, the researchers invited health professionals who had completed graduate master’s courses or had more than five years of experience in related fields.

To increase the diversity of the expert panel, we targeted Korean professionals with recognized expertise in lifestyle and/or healthcare management within the scope of occupational therapy, physical therapy, nursing, and nutrition. The eligibility criteria were as follows: (1) ability to complete approximately three rounds of surveys within two months, (2) no restrictions on computer skills, and (3) no restrictions on access to the Internet and e-mail.

After obtaining consent for participation, a Delphi survey was conducted. For those selected as potential experts, an invitation email was sent that included a letter introducing the Delphi survey, a consent form related to the study, and an IRB guide. The experts were informed that they had to respond to all rounds until a consensus was reached (approximately three rounds), each round was given an appropriate amount of time to respond, participation was voluntary, and they could withdraw at any time. They were also informed that their individual responses would not be shared with other expert panels and their contributions would be kept confidential. Delphi surveys were conducted only with experts who expressed consent, and as a reward for their time, participants were given a $50 gift card after completing all the Delphi surveys.

### The Delphi method

The study was conducted with participants who provided consent to potential expert panels. Demographic information was collected from the participants. Participants were instructed to read the guidelines introducing the concept of multi-faceted lifestyle components developed to create lifestyle assessment tools in the introduction of the survey attached to the email (Appendix 1). The first section explains the lifestyle factors of physical activity, eating habits, activity participation, and activity diversity, based on a literature review and previous studies. Participants were then shown the following sections consisting of 72 questions:18 areas of physical activity, 18 areas of nutrition, 18 areas of social relationships, and 18 areas of social participation. These items were developed based on literature review [9,23–26].

**Fig 1:**
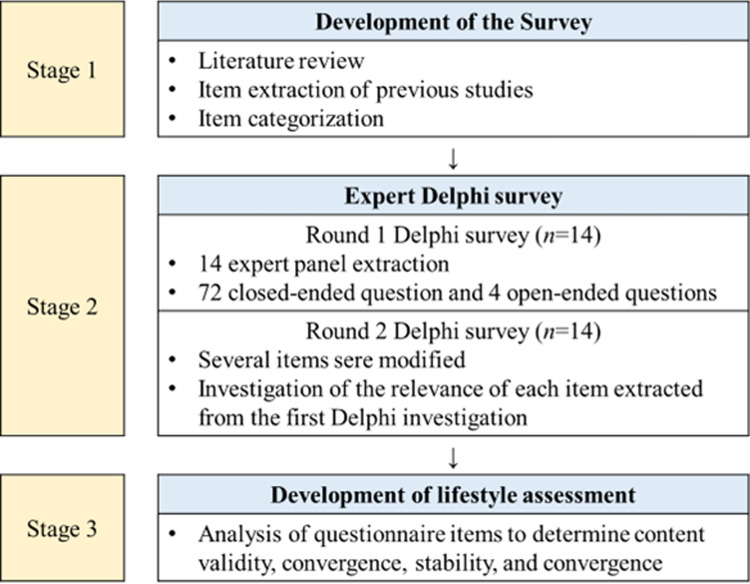
Research process.

The Delphi survey consisted of three steps (Fig 1). In the first stage, a literature review of the development of the questions was derived from previous studies. In the second stage, the Delphi was conducted with experts, and the Delphi had a total of two rounds. The research team provided information about the study and obtained consent from all participants from a panel of experts before the study began. The participants responded to the first- and second-round questionnaires delivered through e-mail. Each round of the survey was conducted for one week, and to maximize the response rate, participants who did not respond were given additional time to send a reminder and answer a day after the deadline. In the final stage, active, balanced, connected, diversity (ABCD) was developed that reflected the opinions of experts.

### Round 1

The survey was open and closed to accommodate the opinions of the expert panel. The first survey consisted of 72 closed-ended and four open-ended questions. Each participant was encouraged to submit their revised recommendations and additional comments regarding the questionnaire. Round 1 of the survey took approximately 30 minutes to complete.

### Round 2

**Fig 2:**
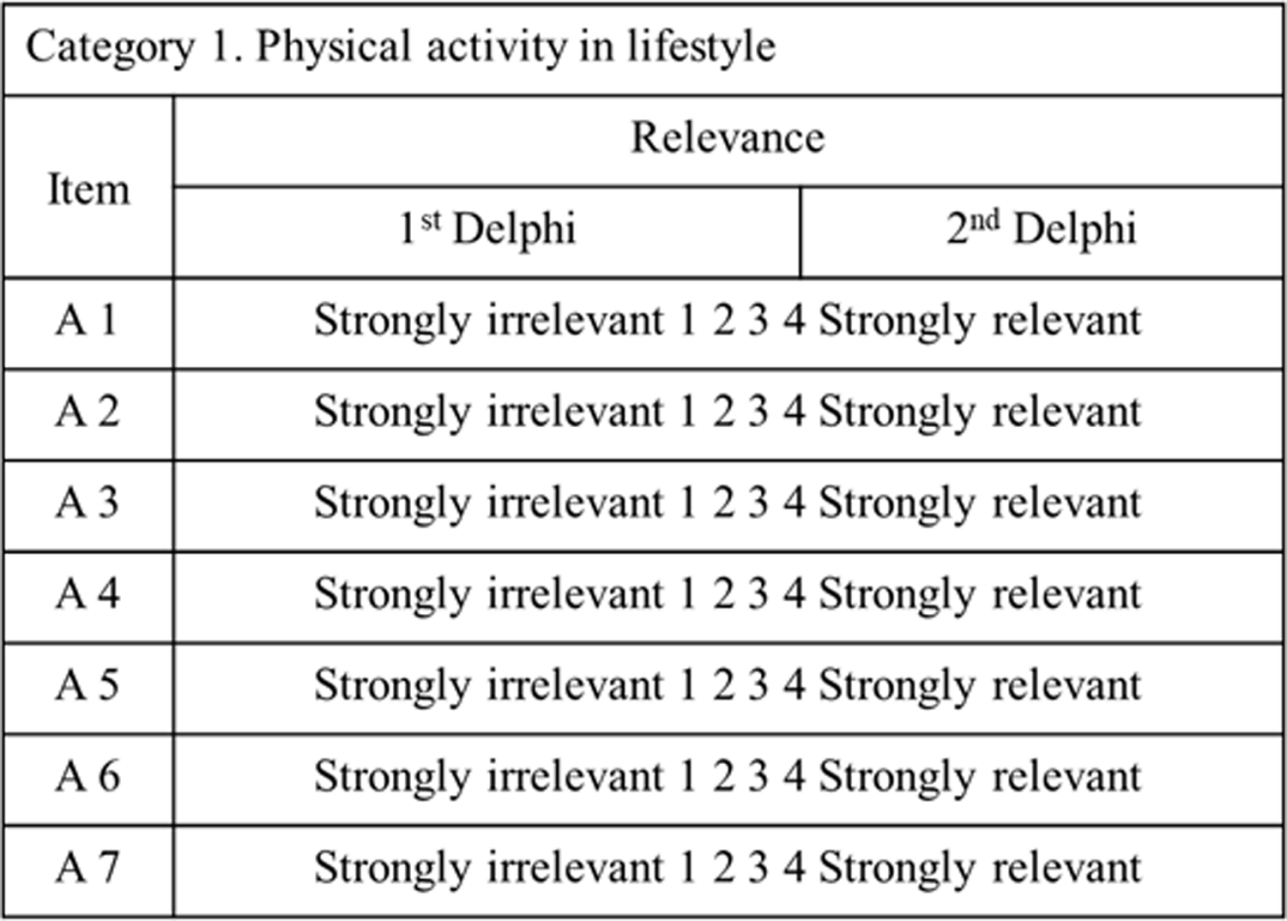
Examples assess the relevance of each item.

The Round 2 survey was revised to reflect the responses of the expert panels in Round 1. It consisted of 72 closed-ended questions in four domains (physical activity, nutrition, social relationships, and social participation). Each participant was informed via e-mail and asked to answer each question using a 4-point Likert scale (strongly irrelevant, not relevant, relevant, or strongly relevant). Neutral (ordinary) scores were not included (Fig 2) to obtain clear opinions from the expert panel. In the second round, four items from the first round were modified.

### Data analysis

**Fig 3:**
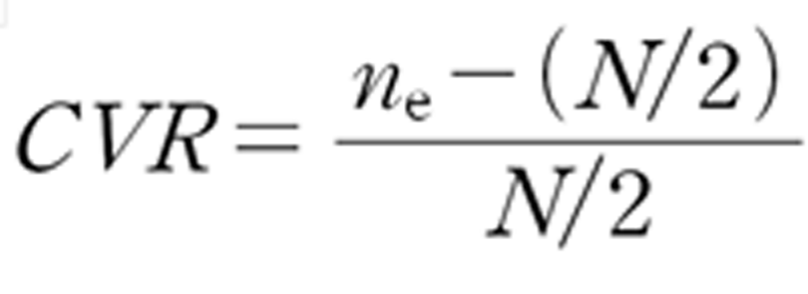
Formulation of CVR (content validity ratio).

To derive opinions, the collected response data were analyzed for means, standard deviations, medians, content validity, convergence, and stability. For general information from the expert panel, the mean, standard deviation, and median were obtained using descriptive statistics. The convergence, stability, and consensus for the collected values were calculated to confirm the validity of the item content. The degree of convergence has a value of zero when all opinions converge at a point and increases when opinion deviations are large. This means that closer to zero, the more valid the item [23–8]. The calculation formula for the CVR value is as follows (Fig 3):

During repeated survey, the smaller the response difference of the panel, the higher the stability if the response is constant, the higher the stability if the response is 0.5 ~ 0.8, and the additional investigation is required if it is 0.8 or more [27], The degree of agreement has a value of 1 when the opinion is congruent, and the closer to 1, the more valid the item [28]. For the fitness value of each item, the content validity ratio (CVR) value was analyzed based on the criteria of Lawshe (1975) [29], and the data were analyzed based on 0.51, the minimum value according to the 14 expert panels participating in this study.

## Results

### Demographics of the expert panels

Sixteen Delphi expert invitations were sent to potential participants. Of these, 14 participants responded by expressing their consent to participate. A panel of 14 experts who participated in this study completed the first and second surveys. The expert panel that participated in this study had a 100% response rate and no participants dropped out. The demographic characteristics of the participants are shown in Table 1. Seven (50%) participants were female and seven (50%) were male. They had an average of 8.86 years of experience in their major fields of expertise. The expert panel comprised various health experts in nursing, nutrition, occupational therapy, and physical therapy.

**Table 1:** Demographics of the expert panels.

		Round 1 (%)	Round 2 (%)
Sample		14	14
Response rate (%)		100	100
Gender	Male	7 (50.00)	7 (50.00)
	Female	7 (50.00)	7 (50.00)
Work experience	1 ~ 3 years	3 (21.43)	3 (21.43)
	4 ~ 6 years	2 (14.29)	2 (14.29)
	7 ~ 9 years	5 (35.71)	5 (35.71)
	10 ≤ years	4 (28.57)	4 (28.57)
	Mean ± SD	8.86 ± 7.02	
Major field of study	Nursing	4 (28.57)	4 (28.57)
	Nutrition	4 (28.57)	4 (28.57)
	Occupational therapy	3 (21.43)	3 (21.43)
	Physical therapy	3 (21.43)	3 (21.43)

### Results of round 1

The categories and items of the detailed results of the Delphi Round 1 are presented in Table 2. Fourteen experts responded to all questions in the first-round survey, and the first round consisted of 76 questions, including 72 closed-ended questions and 4 open-ended questions in four categories. In the physical activity category, the range of the CVR ratio was 0.57 ~  1.00, and content validity was verified for all items. In the nutrition category, among the imbalanced items, the CVR ratio of two items asking about eating speed, drinking, and smoking was 0.43, falling short of the standard value. In the case of the social relations and social participation categories, the CVR ratio of each item was 0.43, and consensus was not reached. Four questions for which a consensus was not reached in the first Delphi survey were modified according to the opinions of experts written in open-ended questions, and then a second Delphi was conducted.

**Table 2:** Lifestyle assessment in the round 1st survey.

Category	Items	Sub-items	Mean*	SD**	CVR***
Physical activity	Active	I regularly do strength/aerobic exercise at least 3 days a week.	3.71	0.45	1.00
		I do high-intensity exercise that gets me out of breath at least once a week.	3.71	0.45	1.00
		I walk 20 minutes a day including moving.	3.54	0.50	1.00
		I move a lot in my daily life.	3.31	0.72	0.71
		I prefer public transportation or walking.	3.14	0.83	0.71
		I usually do light exercises such as stretching and gymnastics with my bare hands on a regular basis.	3.64	0.61	0.86
		I’m not regular, but I exercise.	3.00	0.76	0.71
		I’m taking a class to learn exercise.	3.14	1.06	0.57
		I exercise with a regular plan.	3.57	0.49	1.00
	Sedentary	I don’t have a separate exercise time that I do regularly or planned.	3.29	0.88	0.86
		I don’t do any physical activity other than walking daily.	3.43	0.90	0.71
		I use my car rather than public transportation.	3.00	1.13	0.57
		I use transportation instead of walking whenever possible.	3.29	0.70	0.71
		I spend my leisure time sitting rather than doing leisure activities that involve physical activity.	3.57	0.62	0.86
		I sit or lie down most of the day.	3.92	0.61	0.86
		I don’t move if whenever possible.	3.29	0.80	0.57
		I don’t take the time to exercise separately.	3.29	1.03	0.71
		I consider myself not getting enough physical activity.	3.36	0.89	0.71
Nutrition	Balanced	I eat red meat at least twice a week.	3.14	1.06	0.57
		I eat foods containing calcium (milk, cheese, yogurt, cabbage, etc.) once a week.	3.64	0.81	0.86
		I eat raw or cooked vegetables every other day.	3.86	0.35	1.00
		I eat protein (fish, meat, eggs, beans, tofu, etc.) at least twice a day.	3.79	0.41	1.00
		I drink more than 8 cups of water a day.	3.57	0.62	0.86
		I eat nuts every day.	3.21	0.94	0.57
		I eat regularly.	3.71	0.45	1.00
		I eat as evenly as possible.	3.50	0.50	1.00
		I avoid overconsumption of salt.	3.43	0.73	0.71
	Unbalanced	There are many days when I eat processed foods (processed meat, ramen, snacks, drinks, etc.) more than once a day.	3.50	0.91	0.71
		I do not eat foods that contain calcium (milk, cheese, yogurt, cabbage, etc.).	3.36	0.89	0.71
		I eat vegetables no more than 3 times a week.	3.50	0.82	0.86
		I often don’t eat protein (fish, meat, eggs, beans, tofu, etc.) once a day.	3.29	1.03	0.71
		When I eat with others, I always eat faster than other people.	3.07	1.10	0.43
		I eat late-night snacks at least 3 times a week.	3.21	1.01	0.71
		I eat irregularly.	3.50	0.82	0.86
		I currently smoke or drink alcohol.	2.93	1.16	0.43
		There are many days when I don’t eat two meals a day.	3.21	1.01	0.71
Social relationships	Connected	I live with my family or other people.	3.71	0.59	0.86
		I have enough social interaction with others as much as I want.	3.64	0.48	1.00
		I have close friends (family, relatives, friends) who live close by.	3.79	0.41	1.00
		I have close friends that I see at least once every two weeks.	3.71	0.45	1.00
		I have enough communication with others.	3.71	0.45	1.00
		There is an online meeting that I’m actively participating in.	2.93	0.96	0.57
		I always try to meet new people.	3.07	1.03	0.57
		I have 3 or more regular meetings (class reunions, social gatherings, etc.).	3.43	0.82	0.86
		I have a group or organizations I attend regularly.	3.43	1.05	0.71
	Isolated	I live alone without a partner.	3.21	1.15	0.43
		I feel alone or isolated.	3.36	1.04	0.71
		I spend most of my time alone.	3.43	1.05	0.71
		I have no one to help me when I’m in trouble.	3.50	1.05	0.71
		I do not prefer to hang out with or be with other people.	3.36	0.89	0.71
		I don’t have any meetings or organizations that I attend regularly.	3.50	0.63	0.86
		I don’t feel the need to create new relationships.	3.50	0.82	0.86
		I do not feel the need for social belonging.	3.36	0.89	0.71
		I have difficulty communicating online and am not active.	3.43	0.90	0.71
Social participation	Diversity	I’m working for economic activity.	3.36	1.11	0.57
		I watch performances and artistic activities or participate in creative activities.	3.57	0.49	1.00
		I actively participate in community events such as festivals.	3.50	0.91	0.71
		I attend online and offline lecture.	3.57	0.62	0.86
		I actively use community public facilities (libraries, educational facilities, leisure centers, etc.).	3.36	0.97	0.57
		I’m interested in and participate in political activities and voting.	3.64	0.61	0.86
		I’m interested in solving community problems.	3.14	0.91	0.57
		I participate in residents’ self-governing activities.	3.21	0.86	0.71
		I regularly participate in religious activities.	3.14	1.19	0.57
	Monotonous	I’m not working for economic activity.	3.57	0.90	0.71
		I do not voluntarily participate in activities with others.	3.71	0.45	1.00
		I don’t know much information about community events, festivals, etc.	3.43	0.73	0.71
		I don’t have any special events or events in my daily life.	3.36	0.72	0.71
		I’m not interested in political matters or community news.	3.21	1.08	0.57
		My daily life is monotonous and repetitive.	3.64	0.48	1.00
		Rather than trying to change my daily life, I keep it as it is.	3.29	0.88	0.71
		I have restrictions on community participation without the help of others.	2.93	1.03	0.43
		I enjoy listening to music or watching TV alone.	3.57	0.62	0.86

* Mean = average of each sub-items; ^**^SD =  standard deviation of each sub-items; ^***^CVR =  content validity ratio of each sub-items.

### Results of round 2

The detailed results of the Delphi Round 2 are presented in Table 3. Fourteen experts responded to all the questions in the second survey. In the first round, the questionnaire was administered by modifying four items that did not reach consensus because they did not reach the standard CVR ratio. In the physical activity category, content validity was verified for all items, with CVR ratios ranging from 0.57 to 1.00. In the case of nutrition, the two items asking about eating speed, township, and smoking, which were not agreed upon, were corrected. The CVR ratios were 0.86 and 0.57, respectively, confirming their validity. However, there was no consensus on nut consumption, with a score of 0.43. In the social relationship category, the question of whether to live alone was modified to reflect the first open-ended opinion, but the CVR ratio was 0.29; thus, a consensus was not reached. In the social relations category, the CVR ratio of the question asking about regular activity participation was 0.43, which did not reach a consensus, and the question of whether there was help for community participation reached agreement with a CVR ratio of 0.57 through modification reflecting the first open-ended opinion was reached.

**Table 3:** Lifestyle assessment in the round 2nd survey.

Category	Items	Sub-items	Mean*	SD**	CVR***
Physical activity	Active	I regularly do strength/aerobic exercise at least 3 days a week.	3.93	0.26	0.86
		I do high-intensity exercise that gets me out of breath at least once a week.	3.79	0.41	1.00
		I walk 20 minutes a day including moving.	3.93	0.26	1.00
		I move a lot in my daily life.	3.57	0.62	0.86
		I prefer public transportation or walking.	3.07	0.82	0.85
		I usually do light exercises such as stretching and gymnastics with my bare hands on a regular basis.	3.64	0.48	1.00
		I’m not regular, but I exercise.	3.14	0.64	0.71
		I’m taking a class to learn exercise.	3.14	0.74	0.57
		I exercise with a regular plan.	3.57	0.49	1.00
	Sedentary	I don’t have a separate exercise time that I do regularly or planned.	3.57	0.49	1.00
		I don’t do any physical activity other than walking daily.	3.43	0.62	0.86
		I use my car rather than public transportation.	3.00	0.76	0.71
		I use transportation instead of walking whenever possible.	3.14	0.52	0.86
		I spend my leisure time sitting rather than doing leisure activities that involve physical activity.	3.64	0.48	1.00
		I sit or lie down most of the day.	3.36	0.62	0.85
		I don’t move if whenever possible.	3.43	0.62	0.86
		I don’t take the time to exercise separately.	3.29	0.59	0.86
		I consider myself not getting enough physical activity.	3.43	0.62	1.00
Nutrition	Balanced	I eat red meat at least twice a week.	3.29	0.59	0.86
		I eat foods containing calcium (milk, cheese, yogurt, cabbage, etc.) once a week.	3.79	0.41	1.00
		I eat raw or cooked vegetables every other day.	3.79	0.41	1.00
		I eat protein (fish, meat, eggs, beans, tofu, etc.) at least twice a day.	3.86	0.35	1.00
		I drink more than 8 cups of water a day.	3.50	0.63	0.86
		I eat nuts every day.	2.93	0.88	0.43
		I eat regularly.	3.93	0.26	1.00
		I eat as evenly as possible.	3.93	0.26	1.00
		I avoid overconsumption of salt.	3.36	0.61	0.86
	Unbalanced	There are many days when I eat processed foods (processed meat, ramen, snacks, drinks, etc.) more than once a day.	3.50	0.63	0.86
		I do not eat foods that contain calcium (milk, cheese, yogurt, cabbage, etc.).	3.43	0.62	0.86
		I eat vegetables no more than 3 times a week.	3.57	0.49	1.00
		I often don’t eat protein (fish, meat, eggs, beans, tofu, etc.) once a day.	3.36	0.61	0.86
		I tend to eat faster than other people.	3.36	0.61	0.86
		I eat late-night snacks at least 3 times a week.	3.57	0.49	1.00
		I eat irregularly.	3.86	0.35	1.00
		I currently smoke (including all types of tobacco).	3.21	0.94	0.57
		There are many days when I don’t eat two meals a day.	3.43	0.62	0.86
	Connected	I live with my family or other people.	3.71	0.59	0.86
		I have enough social interaction with others as much as I want.	3.71	0.45	1.00
		I have close friends (family, relatives, friends) who live close by.	3.79	0.41	1.00
		I have close friends that I see at least once every two weeks.	3.79	0.41	1.00
		I have enough communication with others.	3.79	0.41	1.00
		There is an online meeting that I’m actively participating in.	3.21	0.56	0.86
		I always try to meet new people.	3.07	0.46	0.86
		I have 3 or more regular meetings (class reunions, social gatherings, etc.).	3.14	0.52	0.86
		I have a group or organizations I attend regularly.	3.57	0.62	0.86
	Isolated	I currently live alone.	3.14	1.06	0.29
		I feel alone or isolated.	3.57	0.73	0.71
		I spend most of my time alone.	3.71	0.59	0.86
		I have no one to help me when I’m in trouble.	3.79	0.56	0.86
		I do not prefer to hang out with or be with other people.	3.50	0.50	1.00
		I don’t have any meetings or organizations that I attend regularly.	3.14	0.64	0.71
		I don’t feel the need to create new relationships.	3.50	0.50	1.00
		I do not feel the need for social belonging.	3.21	0.56	0.86
		I have difficulty communicating online and am not active.	3.21	0.77	0.57
Social participation	Diversity	I’m working for economic activity.	3.21	0.67	0.71
		I watch performances and artistic activities or participate in creative activities.	3.71	0.59	0.86
		I actively participate in community events such as festivals.	3.29	0.80	0.57
		I attend online and offline lecture.	3.14	0.74	0.57
		I actively use community public facilities (libraries, educational facilities, leisure centers, etc.).	3.43	0.73	0.71
		I’m interested in and participate in political activities and voting.	3.36	0.72	0.71
		I’m interested in solving community problems.	3.21	0.67	0.71
		I participate in residents’ self-governing activities.	3.36	0.72	0.71
		I regularly participate in religious activities.	3.00	0.93	0.43
	Monotonous	I’m not working for economic activity.	3.50	0.63	0.86
		I do not voluntarily participate in activities with others.	3.43	0.49	1.00
		I don’t know much information about community events, festivals, etc.	3.07	0.70	0.57
		I don’t have any special events or events in my daily life.	3.43	0.62	0.86
		I’m not interested in political matters or community news.	3.14	0.74	0.57
		My daily life is monotonous and repetitive.	3.50	0.50	1.00
		Rather than trying to change my daily life, I keep it as it is.	3.29	0.59	0.86
		I have limitations (difficulty) in participating in the community by myself.	3.07	0.70	0.57
		I enjoy listening to music or watching TV alone.	3.36	0.72	0.71

* Mean = average of each sub-items; ^**^SD =  standard deviation of each sub-items; ^***^CVR =  content validity ratio of each sub-items.

The second Delphi results showed a relatively low standard deviation and relatively high content validity compared with the first (Table 4).

**Table 4:** Average of the expert panels.

	M^*^	SD^**^	CVR^***^	Convergence	Consensus
1st Delphi	3.83	0.96	0.77	0.50	0.75
2nd Delphi	3.44	0.74	0.83	0.50	0.75

* Mean = average of all sub-items; ^**^SD =  standard deviation of all sub-items; ^***^CVR =  content validity ratio of all sub-items.

## Discussion

The necessity of developing an assessment tool that can identify lifestyle tendencies reflecting the diversity of activities and the intensity of health-related routines and habits repeated in daily life was confirmed. The development of these assessment tools will support the multi-faceted and systematic measurement of an individual’s physical, social, and mental health. The field of health promotion is increasingly emphasizing the importance of evaluating a multi-dimensional and dynamic lifestyle as an approach to shaping and improving a healthy lifestyle [10]. In addition, Studies [30] and [31] reinforce our understanding that healthy lifestyles support physical and mental well-being, with [30] specifically linking lifestyle diversity to successful aging.However, in previous studies, quantifying and analyzing the frequency and exposure of behaviors such as physical activity, smoking, and drinking had limitations in measuring aspects of habit, behavior, management, and diversity [11]. Accordingly, we decided to develop a comprehensive evaluation tool that could evaluate an individual’s lifestyle in a multi-faceted and systematic manner by applying the Delphi technique. In this study, we discuss our proposal based on the consensus of an expert panel.

Previous studies have shown that physical activity can be considered a lifestyle factor [32]. Most national and international physical activity guidelines and statements recommend regular physical activity to prevent diseases, promote health, and delay loss [33]. However, according to the expert panel, it was necessary to consider aspects of habits, behaviors, management, and variety and to identify individual preferences or tendencies, including quantified measures such as high-intensity, moderate-intensity, and low-intensity activities. Previous studies have reported that maintaining a healthy lifestyle increases a chronic disease-free lifespan and improves overall physical and emotional well-being [34,35]. One study [34] highlights the role of exercise in reducing chronic disease risks, while another [35] links lifestyle adherence with longer disease-free years. Conversely, being overweight or obese is associated with chronic illness and reduced quality of life [36]. This suggests that regular physical activity is necessary to improve health and quality of life. Therefore, when evaluating an individual’s multi-faceted lifestyle, it is necessary to assess various types of physical activities and habits.

Nutrition is described as a healthy lifestyle factor along with eating habits [32]. This study found that it is necessary to identify unbalanced eating habits, including balanced eating habits. Despite the CVR value of 0.43, nut intake was retained due to its established importance in studies on cardiovascular and immune health [37–40]. Adequate nutrition is critical for the optimal functioning of the body and for strengthening immune function, and nutritional imbalances can adversely affect the immune system [41]. Additionally, adequate nutrition, physical activity, and proper eating habits can reduce the risk of chronic diseases and improve the immune system [42]. A balanced diet that includes fruits, vegetables, whole grains, plant and animal proteins, and healthy fat is a good way to promote better health and proper functioning of the immune system [43]. A balanced diet cannot control infections but can improve the immune system and mental health [41] Therefore, when evaluating a multi-faceted lifestyle, it is necessary to evaluate balanced nutritional status and eating habits.

Social relationships are important factors in physical and psychological health [44]. Individual social relationships are a major factor in mental health, including depression and anxiety. Previous studies have reported that positive interactions with others and maintaining supportive relationships have a positive effect on physical and psychological health [45,46]. This study confirms that it is necessary to ask questions about the physical and human resources that have positive and negative effects on social relationships. However, the question of whether to live alone was 0.29 based on the second round CVR ratio, and a standard value was not reached. It was difficult to determine whether a person lived alone because the type of residence had changed owing to the rapid increase in single-person households in Korea. However, this question was not addressed in this study. Previous studies have reported that social relationships are divided into intra-family and non-family relationships and that objective characteristics of situations, such as the number of social contacts or proximity, are the characteristics of social relationships [47]. In addition, social relationships should consider the volume of relationships (how many social ties are nearby, available, and dependable) and the emotional volume (the degree of intimacy, understanding, and interest shared with others) [47]. Therefore, the question was not deleted, but it is necessary to conduct additional research on the factors of social relationships according to the presence or absence of single-person households in the future by reflecting the additional opinions of experts.

Social participation plays an important role in the quality of life as we age [48]. Social participation is defined as engaging in community activities that provide social interaction [49]. Social participation is an important factor in determining healthy aging [50]. Age, gender, health status, neighborhood, and social environment have been reported as additional factors [51–54]. that have complex relationships with social participation [55]. Similarly, the results of this study confirmed that questions on social participation require consideration of various factors. However, for the presence or absence of participation in religious activities, the second-round CVR was 0.43, and a reference value was not reached. This is because they do not consider non-religious people. However, in previous studies, religious participation has been reported to have potential effects on mental health as an important factor in social participation [56] and to play a positive role in the daily lives of the elderly [57]. Religious participation is closely related to the psychological, physical, and social dimensions of successful aging [58]. Religious participation should be considered when evaluating social participation, but a more subjective understanding of religiosity and participation in religious activities may be questioned. Therefore, the results need to be interpreted cautiously.

The limitations of this study were as follows. The first is the number of Delphi experts. The expert panel comprised a relatively small group of 14 members. Although useful results can be obtained with a panel of 4–11 people [59], there is a limit to generalization, considering that reliability increases as the number of panels increases. Nevertheless, unlike previous studies, this study is meaningful in that a panel of experts in various health-related fields was formed, and conclusions were drawn by collecting various opinions. Second, existing studies were reviewed to develop Delphi questionnaires. It cannot be ruled out that researchers may have missed various theories, backgrounds, and studies in this process. Third, the reliability and validity of the results were analyzed using the opinions of an expert panel. In future, additional studies on the validity and reliability of this questionnaire should be conducted with the public. Finally, the expert panel consisted of only Korean health experts. Therefore, this study reflects only a limited perspective on the Republic of Korea. Therefore, for an international version of this tool, expanding the panel to include experts from multiple countries would provide additional perspectives.

## Conclusion

This study developed a multi-faceted lifestyle assessment tool using a modified Delphi technique. As a result, the 72 items were classified into eight subcategories and four categories. This assessment tool can systematically evaluate lifestyles to achieve health and physical, mental, and social well-being and can be used to identify individual lifestyles necessary for interventions in more depth. However, because the tool was developed in Korea, the possibility that it can be affected by various environmental factors, such as geographical and cultural factors, cannot be ruled out. Therefore, there is a need to conduct research on people living in various communities. Additional research should be conducted to confirm the reliability and validity of the evaluation tools developed.
